# Analysis of whole genome sequences of 16 strains of rubella virus from the United States, 1961–2009

**DOI:** 10.1186/1743-422X-10-32

**Published:** 2013-01-25

**Authors:** Emily Abernathy, Min-hsin Chen, Jayati Bera, Susmita Shrivastava, Ewen Kirkness, Qi Zheng, William Bellini, Joseph Icenogle

**Affiliations:** 1 National Center for Immunizations and Respiratory Diseases, Centers for Disease Control and Prevention, Atlanta, Georgia; 2 J. Craig Venter Institute, Rockville, Maryland, USA

**Keywords:** Rubella virus, Whole genome

## Abstract

Rubella virus is the causative agent of rubella, a mild rash illness, and a potent teratogenic agent when contracted by a pregnant woman. Global rubella control programs target the reduction and elimination of congenital rubella syndrome. Phylogenetic analysis of partial sequences of rubella viruses has contributed to virus surveillance efforts and played an important role in demonstrating that indigenous rubella viruses have been eliminated in the United States. Sixteen wild-type rubella viruses were chosen for whole genome sequencing. All 16 viruses were collected in the United States from 1961 to 2009 and are from 8 of the 13 known rubella genotypes. Phylogenetic analysis of 30 whole genome sequences produced a maximum likelihood tree giving high bootstrap values for all genotypes except provisional genotype 1a. Comparison of the 16 new complete sequences and 14 previously sequenced wild-type viruses found regions with clusters of variable amino acids. The 5^′^ 250 nucleotides of the genome are more conserved than any other part of the genome. Genotype specific deletions in the untranslated region between the non-structural and structural open reading frames were observed for genotypes 2B and genotype 1G. No evidence was seen for recombination events among the 30 viruses. The analysis presented here is consistent with previous reports on the genetic characterization of rubella virus genomes. Conserved and variable regions were identified and additional evidence for genotype specific nucleotide deletions in the intergenic region was found. Phylogenetic analysis confirmed genotype groupings originally based on structural protein coding region sequences, which provides support for the WHO nomenclature for genetic characterization of wild-type rubella viruses.

## Background

Rubella virus (RV) is a positive-polarity, single-stranded RNA virus and the sole member of the *Rubivirus* genus of the *Togaviridae* family. The virus causes a mild childhood disease, but is also a potent teratogenic agent when contracted by a pregnant woman. Therefore, the goal of rubella control and elimination programs is the reduction or elimination of the congenital rubella syndrome (CRS) that occurs in 90% of infants whose mothers were infected with rubella in their first trimester [1]. The genome contains two open reading frames (ORFs), both of which encode precursor proteins that are proteolytically cleaved into functional proteins. The 5^′^ proximal ORF encodes the non-structural proteins (NSP) P150 and P90. The 3^′^ proximal ORF encodes the structural proteins (SP), the capsid (C) and two glycoproteins, E2 and E1. There are 3 untranslated regions (UTRs) in the rubella virus genome: a 40-nucleotide (nt) sequence at the 5^′^ terminus, an approximately 120-nt intergenic region (IR) between the two ORFs, and a 59-nt region at the 3^′^ terminus.

There are currently whole genome sequences for 21 RVs in Genbank (there are multiple sequences for some viruses, e.g. the vaccine strain RA27/3). Almost half of the sequences (11/21) are from wild-type and vaccine viruses from the 1960^′^s, a decade when a world-wide epidemic of rubella occurred, resulting in 20,000 cases of CRS in the United States alone [2]. Vaccination against rubella was initiated in the 1970^′^s, mainly in industrialized countries. No further global epidemics were recorded and interest in collecting and characterizing rubella viruses waned. As a result, very few virus isolates exist from the 1970^′^s, 1980^′^s and the early 1990^′^s. In the 1990^′^s efforts to improve rubella control were strengthened globally as evidenced by an increase in the number of countries including rubella-containing vaccine in their national immunization programs (from 43% of WHO member states in 1996 to 67% of member states in 2009) [3].

The use of molecular epidemiology was recognized as a powerful tool for virus surveillance and isolations of RVs to be used for this purpose greatly increased in the late 1990^′^s and 2000^′^s. In 2005, a systematic nomenclature was adopted by the WHO [4,5]. Briefly, genetic characterization has identified 2 clades which differ by 8-10% at the nt level. Clade 1 is divided into 10 genotypes (1a, 1B, 1C, 1D, 1E, 1F, 1G, 1h, 1i, and 1j), of which 6 are recognized and 4 are provisional (designated by lower case letters). Clade 2 contains 3 genotypes (2A, 2B, and 2C). Rubella virus was declared eliminated from the United States in 2005 [6] and molecular epidemiological analysis of rubella viruses was used to demonstrate that indigenous rubella virus had been eliminated from the country [7]. Imported cases of rubella into the United States continue to occur and efforts are made to obtain viruses for molecular surveillance. Although sequence analysis of a 739-nt region in the E1 coding region has been determined sufficient for phylogenetic analysis [4,5], obtaining the sequences of the whole viral genome can contribute to a greater understanding of the virus. This work adds 16 complete wild-type rubella virus sequences with an emphasis on recent isolates from currently circulating genotypes. An initial analysis of these virus sequences is provided here.

## Results

### Phylogenic analysis of 30 viruses

**Figure 1:**
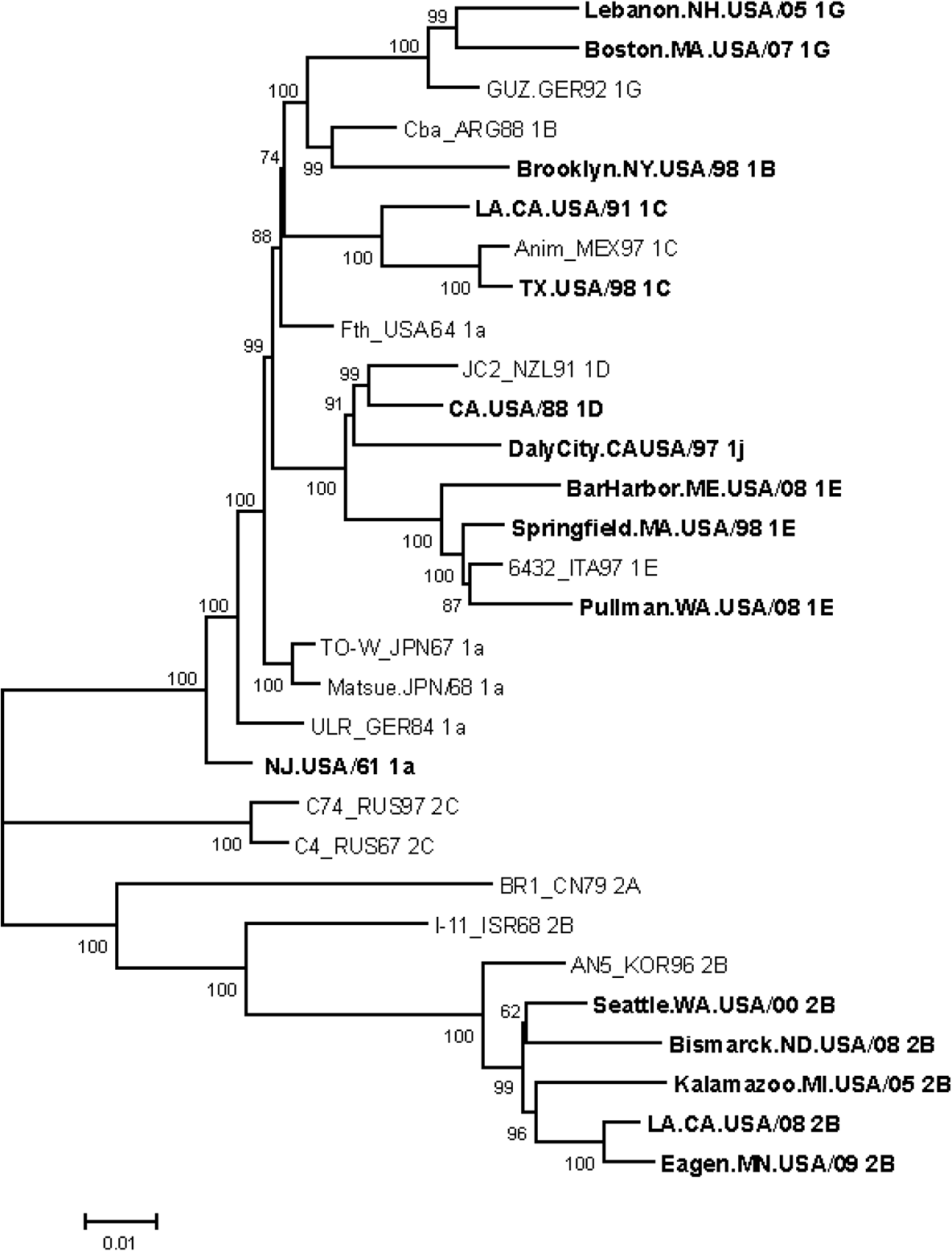
**Phylogenetic tree of the 30 viruses. **The first 21 nts were deleted from the sequence alignment due to a gap in one sequence. Baysian Information Criterion (BIC) scores for different models were computed using the MEGA 5.05 program with the default settings. TN93 with a proportional discrete Gamma distribution (+G) and a fraction of invariant sites (+I), was selected as best fitted model with the lowest BIC scores. The transition/transversion bias for TN93 + G + I model was estimated to be 6.85, using MEGA 5.05 under the Kimura 2-parameter model. The maximum likelihood tree constructed by MEGA 5.05 program with default settings and TN93 + G + I model is shown.

The 16 new genomic sequences (without the 5^′^ 21 nts) were compared with 14 wild-type genomic sequences available from Genbank (Table 1). The TN93 + G + I model was selected as the best fitted model using the MEGA 5.05 program [8] and a maximum likelihood tree constructed using this model is shown in Figure 1. Bootstrap values for the 10 genotypes represented on the tree (there were no whole genome sequences available for genotypes 1h, 1i, and 1F) are greater than 90% for all but provisional genotype 1a. It has previously been noted that genotype 1a viruses do not reliably cluster into a single group, but groupings of this genotype are still distinct from viruses of other genotypes [5]. Consistent with previous findings, pairwise comparisons using the Maximum Composite Likelihood model [8] and 9721 nts found the mean genetic distance between Clades 1 and 2 to be 9% for the 30 viruses; additional analysis of sequence variation in regions of the genomes is included below.

**Table 1:** List of rubella viruses used in this study

Virus*	**Genotype**	**Isolation site, year**	**Accession No.**	**Source^/ Reference**	**Passage^ History**
**NJ.USA/61**	**1a**	**New Jersey, USA, 1961**	**JN635281**	**ATCC**	**Vero 2**
**Brooklyn.NY.USA/98**	**1B**	**New York, USA, 1998**	**JN635282**	**Venezuela**	**Vero 4**
**LA.CA.USA/91**	**1C**	**California, USA, 1991**	**JN635283**		**Vero 3**
**TX.USA/98**	**1C**	**Texas, USA, 1998**	**JN635284**		**Vero 2**
**CA.USA/88**	**1D**	**California, USA, 1988**	**JN635285**		**AGMK 2, BSC 2, Vero 2**
**BarHarbor.ME.USA/08**	**1E**	**Maine, USA, 2008**	**JN635286**	**Cruise ship**	**Vero 3**
**Springfield.MA.USA/98**	**1E**	**Massachusetts, USA, 1998**	**JN635287**	**Ukraine**	**Vero 4**
**Pullman.WA.USA/08**	**1E**	**Washington, USA, 2008**	**JN635288**	**China**	**Vero 4**
**Boston.MA.USA/07**	**1 G**	**Massachusetts, USA, 2007**	**JN635289**	**Uganda**	**Vero 4**
**Lebanon.NH.USA/05**	**1 G**	**New Hampshire, USA, 2005**	**JN635290**	**Cote d’Ivoire**	**Vero 4**
**DalyCity.CA.USA/97**	**1j**	**California, USA, 1997**	**JN635291**	**Philippines**	**RK13 1, BSC 1, Vero 3**
**Kalamazoo.MI.USA/07**	**2B**	**Michigan, USA, 2007**	**JN635292**		**Vero 3**
**Seattle.WA.USA/00**	**2B**	**Washington, USA, 2000**	**JN635293**	**India**	**Vero 4**
**LA.CA.USA/08**	**2B**	**California, USA, 2008**	**JN635294**	**India**	**Vero 5**
**Eagen.MN.USA/09**	**2B**	**Minnesota, USA, 2009**	**JN635295**		**Vero 4**
**Bismarck.ND.USA/08**	**2B**	**North Dakota, USA, 2008**	**JN635296**	**India**	**Vero 3**
F-Th_USA64	1a	Connecticut, USA, 1964	M15240	[9]	NA
ULR_GER84	1a	Leipzig, Germany, 1984	AF435865	[10]	NA
TO-W_JAP67	1a	Toyama, Japan, 1967	AB047330	[11]	NA
Matsue.JPN/68	1a	Matsue, Japan, 1968	AB222609	[12]	NA
Cba_ARG88	1B	Cordoba, Argentina, 1988	DQ085339	[9]	NA
Anim_MEX97	1C	Baja California, Mexico, 1997	DQ085341	[9]	NA
JC2_NZL91	1D	Auckland, New Zealand, 1991	DQ388281	[9]	NA
6423_ITA97	1E	Pavia, Italy, 1997	DQ085343	[9]	NA
GUZ_GER92	1 G	Stuttgart, Germany, 1992	DQ388280	[9]	NA
BR1-CN79	2A	Beijing, China, 1979	AY258322	[13]	NA
AN5_KOR96	2B	Seoul, South Korea, 1996	DQ085342	[9]	NA
I-11_ISR68	2B	Tel Aviv, Israel, 1968	DQ085338	[9]	NA
C4_RUS67	2C	Moscow, Russia, 1967	DQ388279	[9]	NA
C74_RUS97	2C	Moscow, Russia, 1997	DQ085340	[9]	NA

* The 16 new sequences are in bold.

^ The epidemiological link (source) and passage numbers of the 16 viruses are given when known.

### Clusters of variable amino acids and hyper-variable regions (HVR)

**Figure 2:**
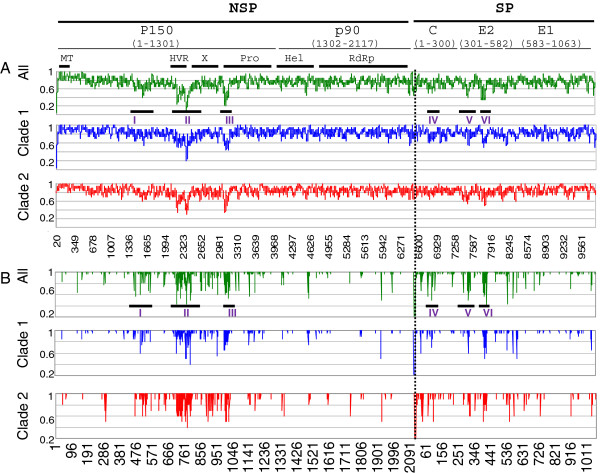
**Identity plots of nucleotide (A) and amino acid (B) sequences of 30 rubella viruses. **The genes and putative domains are shown at the top of the panels. This includes: the methyltransferase (MT), hypervariable region (HVR), X-domain (X), the protease (Pro), helicase (Hel) and RNA-dependent RNA polymerase (RdRp) in NSP and the nucleocapid (C), membrane glycoprotein 2 (E2) and membrane glycoprotein 1 (E1) in SP. The nt analysis was done by counting the number of identical residues at the specific positions of all (green), clade 1 (blue) or clade 2 (red) viruses using Microsoft Office Excel. Comparisons were done using the consensus sequence from all 30 viruses or the clade-specific consensus sequences. Thus, any position at which each virus contains identical nt or aa residues will be 1. The nucleotide identity was plotted using a sliding 30-nt window; data are plotted as moving averages of the number of nucleotide changes. Each line in the amino acid identity plot represents the amount of amino acid identity at the indicated position.

**Figure 3:**
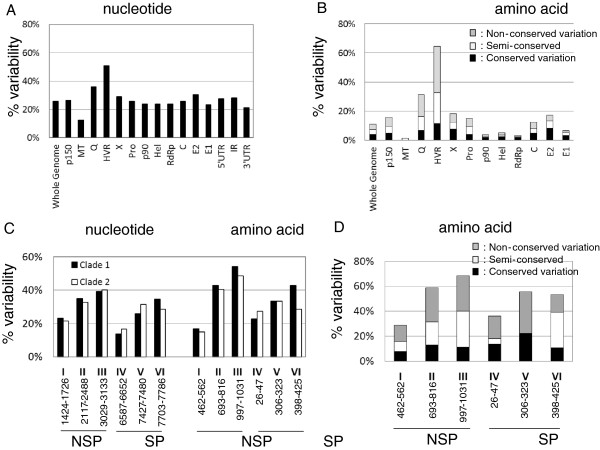
**Sequence variation among 30 rubella viruses. **The percentage of nucleotide variability (**A**) and amino acid variability (**B**) in each domain, as denoted on the X-axis, of 30 rubella viruses relative to overall consensus sequence was calculated using Microsoft Office Excel. The Y-axis indicates the percentage of variability. The amino acid alignment was determined by ClustalW according to the Gonnet PAM 250 matrix. Panels (**C **and **D) **show the variation of nucleic acid and amino acid sequences for the 6 regions shown in Figure 2. The conserved, semi-conserved, or non-conserved designation of each aa position was enumerated based on the most variable aa that was observed at that position amongst the 30 viruses. The clade-specific variations in nucleic acid sequences are compared using clade-specific consensus sequences. The range of each domain is indicated at the bottom of the graphs.

Comparison of the coding sequences of each of the RV domains showed that 74% of the nucleotide positions and 89% of the amino acid positions were invariant among the 30 viruses. Clade specific substitutions were found (Table 2). Previously, a region with genetic hyper-variability (HVR) in the p150 gene (nt 2120-2440/amino acid (aa) 697–800) was identified [10]. Consistent with the previous reports [9,10], the HVR contains significantly higher variability (>50%) than other domains/genes (Figures 2 and 3). Analysis of amino acid sequences of all 30 viruses revealed six clusters (I-VI) where viruses were likely to differ from the consensus sequence, mainly located in the middle of p150 (Cluster I to III) and the N-terminal half of SP (Clusters IV to VI) (Figure 2). More than 50% of the residues in Clusters II, III, V, and VI were variable in at least one of the 30 viruses. Cluster I was the least variable among these clusters and was located in a region without defined function. In Cluster II, which overlapped with the previously defined HVR, variation occurred at 49% of the positions. At the amino acid level, 60% of the amino acid positions were variable and half of the variable positions tolerated non-conserved changes. Such high variability was present in both clade 1 and clade 2 viruses. A region encompassing nt 2177 to 2443 (aa 712–801) in Cluster II showed a variability in amino acid sequence of 74%. This region overlapped with a recently identified Q-domain (aa 497 to 803) which can be deleted from the genome with limited effect on the growth of virus [14]. The C-terminus of Q-domain (Q_C_; aa 548–803) possesses 31% amino acid variability while the amino terminus of Q-domain (aa 497–717) was less variable with changes less than 20%. Cluster III began at the junction between the X-domain (X), also known as the Macro domain, and the protease domain (Pro) and ends near the N-terminus of Pro (Figure 2). Cluster III had more variable nucleic and amino acids than Cluster II with 56% and 69% of the residues exhibiting changes in at least one virus, respectively. The critical catalytic dyad, Cys1152 and His1273, of the protease was well preserved among all viruses.

**Table 2:** Clade-specific amino acid variation

	**aa position**	**Clade 1**	**Clade 2**	**Gene (Domain)**	Reference*
		**(n = 20)**	**(n = 10)**		
NSP	464	R	H	P150 (unknown)	
	551	A	T	P150 (Q domain)	[14]
	725	G	D	P150 (HVR)	[10]
	859	T	A	P150 (X)	[9]
	864	A	E/D	P150 (X)	[9]
	1064	S	G	P150 (Protease)	[15]
	1082	T	A	P150 (Protease)	[15]
	1147	L	R/Q	P150 (Protease)	[15]
SP	116	S	T	C (unknown)	
	308	M	I/L	E2 (antigenicity)	[16]
	323	Q	K	E2 (antigenicity)	[16]
	420	A/V	T	E2 (unknown)	
	700	E	D	E1 (unknown)	

* The references include definitions of the domains.

The clusters of variable amino acids in SP were shorter than those in NSP, with lengths about 20 amino acids, and they did not exhibit variability as high as those found in NSP. Cluster IV (aa 26 to 47) overlapped with the major RNA binding domain of the C protein (aa 28–56) [17]. Clusters V and VI were located at the N-terminal half of E2 and overlapped with the putative antigenic domain of E2 (aa 301–416) [16]. Two out of the 10 changes in cluster V were clade-specific, including Met308 (to Ile or Val in clade 2) and Gln323 (to Lys in clade 2) (Table 2). A clade specific change was also found in cluster VI, where Ala420 (or Val) was preserved among all clade 1 viruses, while it was substituted by Thr in clade 2 viruses (Table 2).

### Conserved regions in RV genomes

In addition to clusters of variable amino acids, regions with little amino acid variation were also identified among the 30 viruses. In general, the putative methyltransferase (MT), helicase (Hel) and RNA-dependent RNA polymerase (RdRp) were less variable and E1 was the least variable among the SP. Among these 30 sequences at the nucleotide level, the percentage of variable nt positions (at least one virus differed from the consensus sequence) across the whole genome was about 26%; approximately 23% of the nucleic acids were variable in the Hel, RdRp and E1. The MT, containing 12% variable nucleic acids, was the most conserved region (Figure 3).

When examining the invariant amino acids among the 30 viruses, 26 regions of more than 30 consecutive identical amino acids were found: 5 of them were within the N-terminal 400 amino acids and 7 were in the putative RdRp domain. The majority of the amino acid conserved regions in the SP gene were within the E1 coding region which overlapped with the putative motifs encoding fusion/antigenicity peptides [18-20]. One of these regions in C also encompassed the antigenic/transmembrane domain of C (nt 7196–7318). Interestingly, while these regions possessed identical amino acid sequences, the nucleic acid identity varied from 10% (the N-terminal 41 amino acids of NSP) to 27% (the C-terminal half of C) (Figure 3).

An examination of the nucleic acid sequences of all 26 regions of more than 30 consecutive identical amino acids as well as the 3 UTRs in the 30 viruses revealed several regions with more than 20 consecutive identical nucleotides. Most of them clustered within the 5^′^ 250 nts of the genome. Among these stretches of identical nucleic acid sequences, two clusters were identified within the IR. One of these stretches, nt 6429–6448, encompassed the subgenomic RNA start site, suggesting that this sequence may contain critical regulatory elements for subgenomic RNA synthesis.

### Nucleotide deletions in the junction region

Nine of the 16 new sequences were 9762 nucleotides in length, while the other 7 viruses had either one or two nucleotide deletions in the IR. These deletions confirm data reported in 2007 [9] and provide additional evidence that these deletions are genotype specific. All five of the genotype 2B viruses sequenced in this study (years of collection ranging from 2000 to 2009; Table 1) had a single deletion at nt 6422, as did the 2 older (1968 and 1996) 2B viruses sequenced previously [9]. Thus, all genotype 2B viruses sequenced thus far contain a one nucleotide deletion which appears to be specific for this genotype. In addition, Zhou et al. reported that the two nucleotide deletion (nts 6480 and 6481) was found in five of seven genotype 1B viruses. Based on the updated rubella nomenclature [5], analyses of the phylogenetic groupings of these 5 viruses with other rubella virus sequences showed that two of the five would currently be classified as 1G viruses (Guz_GER92 and I76 ISR 92), two would belong to genotype 1i (Tom_UK_86 and 0754_GER_92), and the last would be an outlier to genotype 1h (4008_ITA_91). The remaining two 1B viruses, Cba_ARG_88 and Fan_UK_78, which did not contain the two nucleotide deletion, remain classified as genotype 1B in the current standard nomenclature. In this report a genotype 1B virus, Brooklyn.NY.USA/98, did not have the two nucleotide deletion while two genotype 1G viruses, Boston.MA.USA/10.07 and Lebanon.NH.USA/3.05, did have the two nucleotide deletion. The 2 nucleotide deletion in the IR sequences was consistently seen in 4 additional 1G and 1 each additional 1i and 1h viruses, while no deletion was observed in 3 additional 1B viruses (author’s unpublished result). In summary, using the current nomenclature system, the 1B viruses do not contain the two nucleotide deletion, but all of the 1G, 1h, and 1i viruses do have the two deleted nucleotides. Therefore, this deletion appears to be specific for genotypes 1G, 1h, and 1i.

### Recombination

To investigate possible recombination events among the 30 rubella whole genome sequences, six different algorithms implemented in the RDP3 program [21], RDP, GENECONV, MaxChi, Chimaera, SiScan and 3Seq, were used for analysis. Since no single program provided optimal performance under all conditions, we looked for consistency of the results from different methods. Only MaxChi and Chimaera showed evidence of possible recombination events among CA.USA/88, JC2_NZL91 and Springfield.MA.USA/98 from nt 715 to 2786 with P-values of 7.764×10^-3^ and 4.989×10^-3^, respectively. Positive data from only two out of six methods were not significant enough to support the conclusion of a recombination event in this region.

## Conclusion

In this study, the whole genome sequences of 30 rubella viruses were analyzed, including the 16 viruses that were sequenced for this report. Although all 16 viruses were isolated from cases within the United States, 10 out of 16 were determined by standard epidemiological means to be importations from other countries. The analysis is consistent with the previous report on the genetic characterization of rubella virus using 19 sequences [9]. The data in this study provides more confidence in the previous analysis by the use of a larger dataset. The phylogenetic analysis also provided high confidence for the current genotype groupings (originally based on structural protein coding region sequences), as evidenced by the high bootstrap values for all genotypes represented except for genotype 1a. Use of the whole genome sequences, thus, provides robust support for the WHO standardized nomenclature for genetic characterization of wild-type rubella viruses.

It was found that 74% of the nucleotides in the 30 genomic sequences were invariant (78% were found previously with a smaller data set [9]) while the amino acid identity was higher at about 89%. Six clusters, located in the coding sequences in the middle of the p150 and the N-terminal half of the SP, were found to contain a higher percentage of variable positions than the rest of the genome. Previously the HVR was identified as the region with the highest local variability; in this study, we identified another region containing even more nucleic and amino acid variability (Cluster III). The nucleotide variabilities found in the coding regions of the E1 and E2 proteins are 23% and 30% respectively, while the amino acid variability is notably different between these two surface proteins, at 7% and 17%, respectively. E1 is believed to be the major immunodominant protein while the role of E2 in immunogenicity has not been fully characterized.

In addition to variable regions, regions containing conserved peptides of more than 30 consecutive identical amino acids were identified among the 30 viruses. The p90 is more conserved than p150 and SP. This includes a region of 114 consecutive identical amino acids in the putative RNA-dependent RNA polymerase domain. The nucleotide sequences coding for these conserved peptides were 80% identical overall; however the nucleotide identity was greater than 90% near the 5^′^ terminus of the genome, while the nucleotide sequence was more variable in the C and E1 coding regions, with only 73% identity. The presence of highly conserved nucleotide sequences suggests their importance in virus replication and may potentially be used as targets for molecular detection of RV. It is not known whether the highly conserved 5^′^ 250-nt region contains critical secondary structures. Two conserved regions in the IR were also noticed which could be critical for the regulation of subgenomic RNA synthesis. Nevertheless, analysis of the 30 sequences also revealed genotype-specific deletions in this region: 6 genotype 2B viruses in this report had a single deletion at 6422 and two 1G viruses had nucleotides 6480 and 6481 deleted, indicating the flexibility of the IR in length. This dinucleotide deletion has also been found in 1h and 1i viruses, providing strong evidence that genotypes G, h, and i diverged from a common ancestor. Unlike two previous reports [9,22], we did not find significant evidence of recombination using the 30 whole genomes and six different programs. Weak possible recombination was predicted close to the 5^′^ termini for three viruses; however, the evidence was not considered significant.

## Methods

Sixteen wild-type rubella virus isolates from the Centers for Disease Control and Prevention (CDC) were chosen for whole genome sequencing at the J. Craig Venter Institute (JCVI) (Table 1). All viruses in this study were collected in the United States, most as a result of routine surveillance, and dates of collection range from 1961 to 2009, with the majority coming from the late 1990^′^s and 2000^′^s. One of the virus isolates is a laboratory strain (NJ.USA/61/1a), commonly known as M33 (ATCC, Manassas, VA) which was the first rubella virus isolate [23]. There are currently 4 entries in GenBank for the M33 strain: X05259 (3382 nts), X72393 (6600 nts), J02620 (1822 nts), and AJ438491 (948 nts). Although the combined X05259 and X72393 sequences cover most of the genome (9749 nts), they contain errors making it difficult to align and use the sequences. Therefore, a resequencing of this historic virus was deemed beneficial. Ten of the isolates were known to have been imported into the United States from other countries, either as acute cases acquired abroad or as CRS cases whose mothers spent time in other countries during the early stages of their pregnancies. Two of the isolates, LA.CA.USA/91/1C and Seattle.WA.USA/16.00/2B, serve as WHO reference viruses (Table 1).

Stocks of the 16 viruses were inoculated into T75 flasks of confluent Vero cells. Five to seven days post-infection, the culture medium was removed and total cellular RNA was extracted using Tri-Reagent (Molecular Research Center, Cincinnati, OH) according to the manufacturer’s protocol. The RNA was resuspended in nuclease-free water and stored at −80°C.

Oligonucleotide primers were designed using an automated primer design tool [24,25]. Clade 1 primers were designed from an alignment of the following reference sequences: 1a_AF435865, 1a_AB222609.1, 1B_DQ085339.1, 1C_DQ085341.1, 1D_DQ388281 and Clade 2 primers were designed from 2A_AY258322.1, 2B_DQ085338.1, and 2C_DQ085340.1. Primers, with M13 tags added, were designed at intervals along both the sense and antisense strands, and provided amplicon coverage of at least 4-fold (Additional file 1: Table S1). RT-PCRs were performed with 1 ng of RNA using OneStep RT-PCR kits (Qiagen, Valencia, CA) according to manufacturer’s instructions with minor modifications. Reactions were scaled down to 1/5 the recommended volumes, the RNA templates were denatured at 95°C for 5 min, and 1.6 U RNase Out (Invitrogen, Carlsbad, CA) was used. The RT-PCR products were sequenced with an ABI Prism BigDye v3.1 terminator cycle sequencing kit (Applied Biosystems, Carlsbad, CA). Raw sequence traces were trimmed to remove any primer-derived sequence as well as low quality sequence, and gene sequences were assembled using Minimus, part of the open-source AMOS project [26]. The gene sequences were then manually edited using ClOE (Closure Editor; JCVI) and ambiguous regions were resolved by additional sequencing when possible. Finally, the Viral Genome ORF Reader [27] was used to check segment lengths, perform alignments, ensure the fidelity of open-reading frames, correlate nt polymorphisms with amino acid changes, and detect any potential sequence errors. The 5^′^ and 3^′^ termini were determined using the 5^′^/3^′^ RACE kit as directed by the manufacturer (Roche Diagnostics, Mannheim, Germany) and oligo dT priming, respectively. The termini sequences were incorporated into the whole genome sequences as described above.

### Sequence and phylogenetic analyses

The sequences were aligned by ClustalW in the MEGA 4.0 software [28]. Phylogenetic analysis was performed using the Mega 5.05 program [8]. The first 21 nts of the 30 sequences were deleted from the sequence alignment due to a gap in one sequence (AF435865). Analysis of variable and conserved positions relative to a consensus sequence was done using Microsoft Excel by comparing alignments of the nt and aa sequences of the 30 viruses. The comparison does not necessarily reflect the evolutionary distance between the viruses. Recombination analysis was performed using the RDP3 program [21].

## Abbreviations

RV: Rubella virus; CRS: Congenital rubella syndrome; ORF: Open reading frame; NSP: Non-structural proteins; SP: Structural proteins; C: Capsid protein; UTR: Un-translated region; nt: Nucleotide; IR: Intergenic region; HVR: Hyper-variable region; aa: Amino acid; X: X-domain; Pro: Protease domain; MT: Methytransferase domain; Hel: Helicase domain; RdRp: RNA dependent RNA polymerase; JCVI: J.Craig Venter Institute; CDC: Centers for Disease Control and Prevention.

## Competing interests

The authors declare that they have no competing interests.

## Authors’ contributions

Virus RNA preparations were done by EA. Sequencing was performed by JB, EK, and EA. Sequence analysis was done by MHC, EA, SS, and QZ. The manuscript was prepared by EA, MHC, WB and JPI. All authors read and approved the final manuscript.

## Authors’ information

The findings and conclusions in this report are those of authors and do not necessarily represent the views of the U.S. Department of Health and Human Services.

## Supplementary Material

Additional file 1: Table S1 Primer sets designed for sequencing of rubella virus clades 1 and 2. Click here for file
