# Supplementary material for: Analysis of whole genome sequences of 16 strains of rubella virus from the United States, 1961–2009
Source: Virol J. 2013 Jan 25;10:32. doi: 10.1186/1743-422X-10-32 (PMC3574052; doi:10.1186/1743-422X-10-32)
Supplement: Additional file 1: Table S1 — Primer sets designed for sequencing of rubella virus clades 1 and 2. [file 1743-422X-10-32-S1.docx]

Supplementary Table 1. Primer sets designed for sequencing of rubella virus clades 1 and 2.

| Clade | Nucleotide numbers* | Forward Primer^ | Reverse Primer^ |
| --- | --- | --- | --- |
| 1 | 1-312 | TGTAAAACGACGGCCAGTCAATGGRARCTAYCGGACCT | CAGGAAACAGCTATGACCACGTGTAGGGCTTCTTTAG |
| 1 | 1036-1381 | TGTAAAACGACGGCCAGTGTGTTCCAGCGCATGCTGTC | CAGGAAACAGCTATGACCCTGTTCCCACTCCTCCATGC |
| 1 | 1122-1462 | TGTAAAACGACGGCCAGTGAGCCACAGCATCACGCTC | CAGGAAACAGCTATGACCCGATTTTGGGGGTTGCAC |
| 1 | 1237-1565 | TGTAAAACGACGGCCAGTAGGTTGGCGGGGATYATGAA | CAGGAAACAGCTATGACCAAGGACGYTCGCGCGGGRC |
| 1 | 1324-1667 | TGTAAAACGACGGCCAGTATYAAGCGGTTCTTCGGCAG | CAGGAAACAGCTATGACCGGAGAGCCTCGAARTCCCAC |
| 1 | 1414-1751 | TGTAAAACGACGGCCAGTCTCGAGGACGGCGGGCRCCA | CAGGAAACAGCTATGACCCGGGGTGGCGGTAGAGCAC |
| 1 | 144-494 | TGTAAAACGACGGCCAGTGGAAGTGCGCGATGTTGTTW | CAGGAAACAGCTATGACCCGTAGAAKGGGCCGCTCG |
| 1 | 1486-1823 | TGTAAAACGACGGCCAGTACCTGGATCGTCCACGCRG | CAGGAAACAGCTATGACCGYGGGTCGCATAAGACCAGG |
| 1 | 1597-1937 | TGTAAAACGACGGCCAGTCTYATCCCGCCGTGGCTGTT | CAGGAAACAGCTATGACCAGGGGCGCTCGGGTGGAG |
| 1 | 1774-2124 | TGTAAAACGACGGCCAGTCTTGAYGAGCCKGGCGAG | CAGGAAACAGCTATGACCAGGTCACGCACCGAGAGYG |
| 1 | 1839-2160 | TGTAAAACGACGGCCAGTGGGCCCYGAACGCCAYTT | CAGGAAACAGCTATGACCGTGACCGCRYTTGCGTCG |
| 1 | 1957-2275 | TGTAAAACGACGGCCAGTGCGTGGGCRAAGTTCTTCC | CAGGAAACAGCTATGACCAGTRCCGCGGGCGTCCRW |
| 1 | 2056-2406 | TGTAAAACGACGGCCAGTCTGATCGCRCTGGCYTTG | CAGGAAACAGCTATGACCACCTCCAGCTCSGCGTSRC |
| 1 | 2106-2456 | TGTAAAACGACGGCCAGTRCTCTCGGTGCGTGACCTG | CAGGAAACAGCTATGACCTGTCTGGGTCTGCCTTGGYT |
| 1 | 2132-2481 | TGTAAAACGACGGCCAGTGTGYAGCGTTCGACGCAAR | CAGGAAACAGCTATGACCCGGGCGTARCTTTCAACGA |
| 1 | 230-574 | TGTAAAACGACGGCCAGTGYGATCACCCAGCACTCCAC | CAGGAAACAGCTATGACCGCACATCTGCATGGGTGTGT |
| 1 | 2338-2682 | TGTAAAACGACGGCCAGTGCGGGTGACYCSGYCCCT | CAGGAAACAGCTATGACCGGTGTSGCCACKGCCTCG |
| 1 | 2484-2806 | TGTAAAACGACGGCCAGTCGCCGGACCCGTGCACCT | CAGGAAACAGCTATGACCYAGCGCGACGATGCTGCG |
| 1 | 2644-2989 | TGTAAAACGACGGCCAGTCTCGCCCCATGCCCCACC | CAGGAAACAGCTATGACCRACGAGCACRGAGGCGTGCG |
| 1 | 2703-3049 | TGTAAAACGACGGCCAGTCCACATCATCCACGCSGTH | CAGGAAACAGCTATGACCGCRRGRTGCGAGGGGCTC |
| 1 | 2896-3239 | TGTAAAACGACGGCCAGTCTYGCGGCTACGCGCRCCGA | CAGGAAACAGCTATGACCTVGCCCAGCTGGTGGCRCCG |
| 1 | 2973-3323 | TGTAAAACGACGGCCAGTCGCCTCYGTGCTCGTYGG | CAGGAAACAGCTATGACCCGGGCTTGAGCACRCTGTA |
| 1 | 3149-3499 | TGTAAAACGACGGCCAGTGCGAACTCTGCCGGTACAC | CAGGAAACAGCTATGACCCCARCAGGTGTTTGGGTCTA |
| 1 | 3279-3617 | TGTAAAACGACGGCCAGTCGCCACGCATTTYCCATTA | CAGGAAACAGCTATGACCAGTCCTCATGRGTKCGGGCT |
| 1 | 3351-3699 | TGTAAAACGACGGCCAGTCGGGAGTGAYATGTGGCG | CAGGAAACAGCTATGACCATCAGGGGGTCGAGGGGRT |
| 1 | 3406-3745 | TGTAAAACGACGGCCAGTACCCCCTCCAACGCYCAC | CAGGAAACAGCTATGACCCTCGGAGCCGACCCACAC |
| 1 | 3546-3873 | TGTAAAACGACGGCCAGTCTACACGAGYGCCGGGTG | CAGGAAACAGCTATGACCACYGCGCAGACGAARTGG |
| 1 | 3588-3925 | TGTAAAACGACGGCCAGTCCTGAGCGAAGCCCGMAC | CAGGAAACAGCTATGACCGACCGCGAGCCAAAGGTG |
| 1 | 3705-4055 | TGTAAAACGACGGCCAGTCGTGGGATGCGCCTGTTC | CAGGAAACAGCTATGACCGTTGGACTGAYGCGAGGGC |
| 1 | 3761-4101 | TGTAAAACGACGGCCAGTACCACCTCCTGGTGTCCCTC | CAGGAAACAGCTATGACCGCGGCCATGTTCCATACYC |
| 1 | 3884-4233 | TGTAAAACGACGGCCAGTCACGCCGCGTCTCGGACC | CAGGAAACAGCTATGACCGCGGCGTTCTTGATGTCGAT |
| 1 | 3948-4285 | TGTAAAACGACGGCCAGTCTGTGCCGCGACCGACGAG | CAGGAAACAGCTATGACCRTAGATGCGGCGGTAGGCG |
| 1 | 4059-4408 | TGTAAAACGACGGCCAGTYCGCAAAGGCCCYTACAAYA | CAGGAAACAGCTATGACCGCGGCAGTTRTTGGCGTART |
| 1 | 4080-4407 | TGTAAAACGACGGCCAGTCAGRGTATGGAACATGGCCG | CAGGAAACAGCTATGACCCGGCAGTTRTTGGCGTARTG |
| 1 | 4109-4448 | TGTAAAACGACGGCCAGTGCAAGACYACCCGCATYCTC | CAGGAAACAGCTATGACCGKCGTGAGCGCTCGGTAG |
| 1 | 418-765 | TGTAAAACGACGGCCAGTGCCGAGACGGCCAGYGAG | CAGGAAACAGCTATGACCGTGCAGGGGCGGGTGTAG |
| 1 | 4249-4585 | TGTAAAACGACGGCCAGTCTGACGAAACCGCTCGCC | CAGGAAACAGCTATGACCCGAGAAGGCGAGGTGRAGGT |
| 1 | 4288-4623 | TGTAAAACGACGGCCAGTGAYGAGGCGTTYACYCTCGG | CAGGAAACAGCTATGACCCGTATGCCRGCYTCGTGR |
| 1 | 4408-4738 | TGTAAAACGACGGCCAGTACCCCCGTCCCYGACCGCT | CAGGAAACAGCTATGACCGCTGACGATGGCGAGGTC |
| 1 | 4450-4798 | TGTAAAACGACGGCCAGTACTTGGCGCTTCCCCGACT | CAGGAAACAGCTATGACCAGCGCGCAGTGAGCCGTC |
| 1 | 4537-4887 | TGTAAAACGACGGCCAGTTGYAACCTYTGGGACGGC | CAGGAAACAGCTATGACCTGYTCRACGGCGACRACG |
| 1 | 4646-4979 | TGTAAAACGACGGCCAGTAGGGYATGAGCGTYGGCAC | CAGGAAACAGCTATGACCCGAAGACGAGCTCCTCCAGR |
| 1 | 4739-5075 | TGTAAAACGACGGCCAGTTGACCCGGGCCTCCGACG | CAGGAAACAGCTATGACCTGTTGAGCAGGTGACGCGAG |
| 1 | 4755-5105 | TGTAAAACGACGGCCAGTCGCACTCTACCTCCACGAGC | CAGGAAACAGCTATGACCGTTCYGTYCCGGGCATCTCG |
| 1 | 4804-5132 | TGTAAAACGACGGCCAGTCTCAGCGCGTTCCTCGAC | CAGGAAACAGCTATGACCGCACGGCGCARACGGCAC |
| 1 | 4932-5279 | TGTAAAACGACGGCCAGTAGACGTGCCGCCCTTCTG | CAGGAAACAGCTATGACCCGATCCGTTCCCGCAAGTA |
| 1 | 4960-5310 | TGTAAAACGACGGCCAGTCTGGAGGAGCTCGTCTTCGG | CAGGAAACAGCTATGACCACGCCCATCTGCGTGTAG |
| 1 | 5053-5397 | TGTAAAACGACGGCCAGTATCTCGCGTCACCTGCTCAA | CAGGAAACAGCTATGACCGGGACGCTCAGGCTCTGG |
| 1 | 5156-5506 | TGTAAAACGACGGCCAGTGGTCGACCCTCCGCACYG | CAGGAAACAGCTATGACCCCAYGCCCGGATCTCAAG |
| 1 | 5241-5574 | TGTAAAACGACGGCCAGTYCAGGAGTGGCGCATGAC | CAGGAAACAGCTATGACCARGGCGCGCATGATGATCT |
| 1 | 532-882 | TGTAAAACGACGGCCAGTGCGGACAGATGCYTGCTYTA | CAGGAAACAGCTATGACCTGGGCAACCTCCCATGAR |
| 1 | 5346-5691 | TGTAAAACGACGGCCAGTCTAYCCYGAGATCTTCGCCG | CAGGAAACAGCTATGACCTGGTTCATGTCGAACTCRGTGA |
| 1 | 5401-5751 | TGTAAAACGACGGCCAGTTTCCTCAARGCCACCYTGAA | CAGGAAACAGCTATGACCCARGGGAGRCCCAAGAGAGC |
| 1 | 5486-5804 | TGTAAAACGACGGCCAGTGCCTTGAGATCCGGGCRT | CAGGAAACAGCTATGACCGTTCGCGCAGGGTGCAGTAG |
| 1 | 5506-5830 | TGTAAAACGACGGCCAGTGCCAAGGAGTGGGTWCAGGT | CAGGAAACAGCTATGACCCTCGCAGCCGGTCTCAGT |
| 1 | 5640-5949 | TGTAAAACGACGGCCAGTYTACACCACCAAYGCCATCG | CAGGAAACAGCTATGACCACCATRTCGTCRCCCTGGAA |
| 1 | 5746-6096 | TGTAAAACGACGGCCAGTCCYTGCGCCGAAGACTACC | CAGGAAACAGCTATGACCAAGAGGCCGGCCGCRGTG |
| 1 | 5896-6245 | TGTAAAACGACGGCCAGTATGGTCCCCAAAGGCGTG | CAGGAAACAGCTATGACCCGTAGTACGCAGCATTGGCR |
| 1 | 6046-6389 | TGTAAAACGACGGCCAGTACCCCHACCCCCAGYTTC | CAGGAAACAGCTATGACCAGTCAGCGTCGTGGAGRTTG |
| 1 | 604-934 | TGTAAAACGACGGCCAGTCTCGTGGCGGTTGACTTGT | CAGGAAACAGCTATGACCGATRGGGAGGGTGCATCG |
| 1 | 6157-6497 | TGTAAAACGACGGCCAGTGAAGAACAGCAGGTGGCCCT | CAGGAAACAGCTATGACCGCTCTCCCGARTGGCAARA |
| 1 | 6334-6661 | TGTAAAACGACGGCCAGTGCGCTCGAGGAGATYCAGAC | CAGGAAACAGCTATGACCRTCTCCGGAGGTGCTGGAGT |
| 1 | 6376-6721 | TGTAAAACGACGGCCAGTCACGACGCTGACTAACGCCC | CAGGAAACAGCTATGACCCTGGCCACGGCCCCGGTT |
| 1 | 6503-6843 | TGTAAAACGACGGCCAGTGTGCCCGAATGGCTTCYA | CAGGAAACAGCTATGACCGTYTGCATRCGCGGGGGTT |
| 1 | 6556-6903 | TGTAAAACGACGGCCAGTCTCGAGGCACAATCMCGC | CAGGAAACAGCTATGACCGCYTGGAACGGGTTGGTC |
| 1 | 6722-7048 | TGTAAAACGACGGCCAGTGYAGGGACTGGTCCAGGG | CAGGAAACAGCTATGACCGGTGCCCAGGTTGGTGAA |
| 1 | 6758-7097 | TGTAAAACGACGGCCAGTAGCGGCAAGAAAGYCGCTC | CAGGAAACAGCTATGACCGGTTGTACATGAGCGCAGGG |
| 1 | 6824-7169 | TGTAAAACGACGGCCAGTAACCCCCGCGYATGCARA | CAGGAAACAGCTATGACCAAACGCCCCTGACGTCGC |
| 1 | 7007-7357 | TGTAAAACGACGGCCAGTTCTTYTACCGCGTCGACCTR | CAGGAAACAGCTATGACCCTGGGGRGCRCCRAAGCG |
| 1 | 7028-7378 | TGTAAAACGACGGCCAGTAYTTCACCAACCTGGGCACC | CAGGAAACAGCTATGACCCAAGAGCCCGGCRAGGAA |
| 1 | 7221-7571 | TGTAAAACGACGGCCAGTGCGCTGGCACCGACTGCT | CAGGAAACAGCTATGACCCGTTTCGGTGRTGCTGGC |
| 1 | 7255-7604 | TGTAAAACGACGGCCAGTGGCCTYGACGGCGACAGC | CAGGAAACAGCTATGACCCTTGGAGCCARTGGCCGGG |
| 1 | 7418-7729 | TGTAAAACGACGGCCAGTAGCCCCGCGCTGAYATGG | CAGGAAACAGCTATGACCGGTRARAGGCGTCGGGGTC |
| 1 | 7467-7811 | TGTAAAACGACGGCCAGTYGCGCACGGGCAGCAYTA | CAGGAAACAGCTATGACCCGCCGCAGCTGTCATTRAG |
| 1 | 7530-7880 | TGTAAAACGACGGCCAGTTCAYGGCGGCACCYTGCG | CAGGAAACAGCTATGACCAGATCARCCGACCGCACC |
| 1 | 7552-7894 | TGTAAAACGACGGCCAGTGGCCAGCAYCACCGAAAC | CAGGAAACAGCTATGACCGGTGGACARCCCRCAGATCA |
| 1 | 7590-7921 | TGTAAAACGACGGCCAGTCCAYTGGCTCCAAGGCGG | CAGGAAACAGCTATGACCRAACCGGGTWGGCGGGTA |
| 1 | 7711-8046 | TGTAAAACGACGGCCAGTACCCCGACGCCTYTYACC | CAGGAAACAGCTATGACCCTCACCAGTTCGGGGCAG |
| 1 | 773-1111 | TGTAAAACGACGGCCAGTTCTACCARGTCYTGCCGGAY | CAGGAAACAGCTATGACCRAACTTGAACACRCGCTCGC |
| 1 | 7853-8180 | TGTAAAACGACGGCCAGTCYGACACCCGGTGCGGTC | CAGGAAACAGCTATGACCGGCGGCACACCATGAATATC |
| 1 | 7923-8271 | TGTAAAACGACGGCCAGTCTGCGYYATGCGGTGGGG | CAGGAAACAGCTATGACCGTGAARGCCTCCTCGCCRTAG |
| 1 | 8009-8347 | TGTAAAACGACGGCCAGTCCGCCCATCCAGGYACCC | CAGGAAACAGCTATGACCGGACTCRAAGCGGACGCC |
| 1 | 8139-8475 | TGTAAAACGACGGCCAGTCCTGCTGGTCCCGTGGGT | CAGGAAACAGCTATGACCCGCGCGCAAGGGGCTGTG |
| 1 | 8255-8603 | TGTAAAACGACGGCCAGTGCGAGGAGGCYTTCACCTAC | CAGGAAACAGCTATGACCCGGTRGGGTGGTACTGCTTR |
| 1 | 8422-8758 | TGTAAAACGACGGCCAGTGAYGTCTCGTGCGAGGGYTT | CAGGAAACAGCTATGACCCCAGACGGTCCTGGTCTCTG |
| 1 | 8474-8818 | TGTAAAACGACGGCCAGTGCATYTGGAAYGGCACACAG | CAGGAAACAGCTATGACCCGTGTTGCAGAAYGGGTGTT |
| 1 | 848-1194 | TGTAAAACGACGGCCAGTGCGCCGTGGCCGAACTYT | CAGGAAACAGCTATGACCGCGTCTTCCTCGGCGCAC |
| 1 | 8656-8994 | TGTAAAACGACGGCCAGTCCCACCGACACYGTGATGAG | CAGGAAACAGCTATGACCACAAGCCGCGAGCAGTCAG |
| 1 | 8714-9058 | TGTAAAACGACGGCCAGTAGACCGTCCGGGTCAAGTTC | CAGGAAACAGCTATGACCRGTGCGCAGCAGGGGGTC |
| 1 | 8911-9250 | TGTAAAACGACGGCCAGTGGCCTYGGGAGCCCGAAT | CAGGAAACAGCTATGACCCGGGCGAACKGTCTTGAAY |
| 1 | 8969-9315 | TGTAAAACGACGGCCAGTATTCCCCTGACTGCTCGC | CAGGAAACAGCTATGACCCCGCACTGRTARCACCCG |
| 1 | 9113-9461 | TGTAAAACGACGGCCAGTAGTGCGGACTCCACATACGC | CAGGAAACAGCTATGACCCGCAGCTGACYTGGTAGGG |
| 1 | 9278-9624 | TGTAAAACGACGGCCAGTCYCGCAATGTGCGTGTGA | CAGGAAACAGCTATGACCCARAYRGCGCCCAGAGTGA |
| 1 | 9397-9747 | TGTAAAACGACGGCCAGTCCCCCTGGGAAGTTYGTCA | CAGGAAACAGCTATGACCTGCGGGRATCTAGTRGGCTR |
| 2 | 6-328 | TGTAAAACGACGGCCAGTRAGCTAYYGGACCTCGCTT | CAGGAAACAGCTATGACCGCTBGGGTCGATGAGGAC |
| 2 | 1008-1350 | TGTAAAACGACGGCCAGTGTGGCGGTGGTTCAGYCTC | CAGGAAACAGCTATGACCGGCACGCTGCCGAARAAC |
| 2 | 1277-1596 | TGTAAAACGACGGCCAGTCCGACTCCCTGAGCGTGG | CAGGAAACAGCTATGACCGCYTCGTCATCYGGCGGG |
| 2 | 1308-1656 | TGTAAAACGACGGCCAGTCACSATYTGGGACGCCATYA | CAGGAAACAGCTATGACCAARTCCCACKCGCGRCAG |
| 2 | 1544-1869 | TGTAAAACGACGGCCAGTACGTYCCACGCGAACKYC | CAGGAAACAGCTATGACCGCACCGGCRGMGAARTGG |
| 2 | 1603-1946 | TGTAAAACGACGGCCAGTCCRCCGTGGCTGTTYGCSGAGC | CAGGAAACAGCTATGACCCRTCAGCCCAGGGGCGCT |
| 2 | 1774-2083 | TGTAAAACGACGGCCAGTCTTGAYGAGCCSGGCGAR | CAGGAAACAGCTATGACCCAGYGTGCGCARRGCCAG |
| 2 | 179-507 | TGTAAAACGACGGCCAGTGGGCCATCGTAGCYGTGATA | CAGGAAACAGCTATGACCTCCTCKGGGTGGACGTARAA |
| 2 | 1800-2127 | TGTAAAACGACGGCCAGTGGCCCTSGTCYTRTGCGA | CAGGAAACAGCTATGACCGGSAGRTCACGCACCGARAG |
| 2 | 2087-2413 | TGTAAAACGACGGCCAGTAACAGGGGGCCGCYYTGG | CAGGAAACAGCTATGACCGCAGGCRACCTCYRGCTCGG |
| 2 | 2087-2413 | TGTAAAACGACGGCCAGTAACAGGGGGCCGCYYTGG | CAGGAAACAGCTATGACCGCAGGCRACCTCYRGCTCGG |
| 2 | 2142-2481 | TGTAAAACGACGGCCAGTYGACGCHAAYGCGGTCAC | CAGGAAACAGCTATGACCCGGGCGTARCTTTCGACRAT |
| 2 | 2436-2783 | TGTAAAACGACGGCCAGTARCCMGGGCGGACCCRGACA | CAGGAAACAGCTATGACCCGCGYTCGAGCAGSGCYT |
| 2 | 2506-2838 | TGTAAAACGACGGCCAGTGTBCGCRACATYATGGACCC | CAGGAAACAGCTATGACCCACGCRACRCACGYCCAC |
| 2 | 2682-3015 | TGTAAAACGACGGCCAGTCGGCCACGGCTGCGGGTA | CAGGAAACAGCTATGACCACGCGCCTGGCAGCGAGCCC |
| 2 | 2702-3031 | TGTAAAACGACGGCCAGTCRCAYATCATCCACGCDGTC | CAGGAAACAGCTATGACCGGTCGGRGGRGGRCTGAC |
| 2 | 287-633 | TGTAAAACGACGGCCAGTGGGGCCCTAAAGAAGCCCTA | CAGGAAACAGCTATGACCGCGACCGGCCASARGTCR |
| 2 | 2915-3263 | TGTAAAACGACGGCCAGTAGCCCGCCGAGCGYGTRAG | CAGGAAACAGCTATGACCAGACGACCACTTCGGGRATG |
| 2 | 2971-3300 | TGTAAAACGACGGCCAGTCACGCYTCYGTGCTCGTK | CAGGAAACAGCTATGACCTTYARTGGGAAGTGCGCRG |
| 2 | 3146-3496 | TGTAAAACGACGGCCAGTGGTGCGARCTCTGYCGGTA | CAGGAAACAGCTATGACCGCAGGTGTTHGGGTCTYGYTC |
| 2 | 3223-3567 | TGTAAAACGACGGCCAGTGCCACCGGYTGGGCGATG | CAGGAAACAGCTATGACCGGGCACCCRGCRCTCGTGTASGC |
| 2 | 3343-3691 | TGTAAAACGACGGCCAGTGGYATGTGCGGGAGYGAYA | CAGGAAACAGCTATGACCGTCGAGGGGGTCYCCRGT |
| 2 | 3372-3713 | TGTAAAACGACGGCCAGTCCGYGGCTGGCAGGGCAT | CAGGAAACAGCTATGACCAGCCCACGGTCTCCATCAG |
| 2 | 3400-3745 | TGTAAAACGACGGCCAGTCGGTGCACCCCYTCYAAY | CAGGAAACAGCTATGACCCTCRGAGCCGACCCATACG |
| 2 | 3546-3875 | TGTAAAACGACGGCCAGTSTACACGAGYGCYGGGTGC | CAGGAAACAGCTATGACCCKACCGCGCARACAAAGTG |
| 2 | 3588-3925 | TGTAAAACGACGGCCAGTYCTSAGCGAAGCCCGCAC | CAGGAAACAGCTATGACCRACCGCGAGCCARAGGTG |
| 2 | 3672-3995 | TGTAAAACGACGGCCAGTCACYGGRGACCCCCTCGAC | CAGGAAACAGCTATGACCGGTCGTCGTARTACGCYTGG |
| 2 | 3763-4101 | TGTAAAACGACGGCCAGTCACCTYCTGGTGTCCCTCCA | CAGGAAACAGCTATGACCGCGGCCATGTTCCAYACY |
| 2 | 3835-4173 | TGTAAAACGACGGCCAGTGARGGGGGCAACCCCACY | CAGGAAACAGCTATGACCGCGTTRGTGGGGCAGACRTA |
| 2 | 3955-4281 | TGTAAAACGACGGCCAGTGCGACCGACGARGGGCTG | CAGGAAACAGCTATGACCATRCGGCGRTAGGCGGCGAG |
| 2 | 4007-4351 | TGTAAAACGACGGCCAGTGCCTYGGGGACGAYGCCAT | CAGGAAACAGCTATGACCCTCCGCGGTGGTTTGGCT |
| 2 | 4060-4408 | TGTAAAACGACGGCCAGTCGYAARGGCCCCTAYAACATC | CAGGAAACAGCTATGACCGCGGCAGTTGTTRGCGTAGT |
| 2 | 4111-4457 | TGTAAAACGACGGCCAGTAAGACYACYCGCATCCTCGC | CAGGAAACAGCTATGACCGCCARGTGTGGCGTGAGC |
| 2 | 4246-4585 | TGTAAAACGACGGCCAGTGCGCTGACRAAACCGCTC | CAGGAAACAGCTATGACCCGARAAGGCGAGGTGRAGGT |
| 2 | 427-777 | TGTAAAACGACGGCCAGTGCCGGYGAGGCSTGGCAY | CAGGAAACAGCTATGACCTAGATGCGYGTGGTGCAGG |
| 2 | 4297-4623 | TGTAAAACGACGGCCAGTTTCACYCTYGGYGGCGAGTA | CAGGAAACAGCTATGACCCGTATGCCRGCCTCRTGRAG |
| 2 | 4420-4769 | TGTAAAACGACGGCCAGTGACCGCTGGCCYACCGAG | CAGGAAACAGCTATGACCGGAGGTAGAGRGCGTCGGAG |
| 2 | 4458-4796 | TGTAAAACGACGGCCAGTCTTCCCCGAYTGYTGGGC | CAGGAAACAGCTATGACCCGCGCAGTGAGCCGTCYT |
| 2 | 4540-4890 | TGTAAAACGACGGCCAGTAACCTYTGGGACGGYCGC | CAGGAAACAGCTATGACCGCCTGYTCGACGGCRACRAC |
| 2 | 4639-4987 | TGTAAAACGACGGCCAGTGAGGCCCAGGGYATGAGC | CAGGAAACAGCTATGACCGGCRCGGCCGAAGACGAG |
| 2 | 4727-5070 | TGTAAAACGACGGCCAGTCCATCGTCAGYCTSACCCG | CAGGAAACAGCTATGACCAGCAGGTGVCGCGAAATG |
| 2 | 4765-5105 | TGTAAAACGACGGCCAGTCTCCAYGARCTCGARGACGG | CAGGAAACAGCTATGACCGYTCYGTBCCGGGCATCTC |
| 2 | 4806-5155 | TGTAAAACGACGGCCAGTYAGCGCSTTYCTCGACGC | CAGGAAACAGCTATGACCRTCCTCRCCYGCGCGGTA |
| 2 | 4934-5284 | TGTAAAACGACGGCCAGTAYGTGCCGCCCTTYTGYC | CAGGAAACAGCTATGACCRAGGTCGATYCGYTCCCG |
| 2 | 5002-5340 | TGTAAAACGACGGCCAGTGCGGAYCTYAACCGCGTR | CAGGAAACAGCTATGACCGCGTAGCGGTCRGTGAGC |
| 2 | 5106-5431 | TGTAAAACGACGGCCAGTYGTYCTCAGYGCTGTTTGCG | CAGGAAACAGCTATGACCGTCYACGCACTTCARGGTGG |
| 2 | 5193-5535 | TGTAAAACGACGGCCAGTGCGCCCTTTCCGYCARAT | CAGGAAACAGCTATGACCGGGGACATRACCTGAACCCA |
| 2 | 5245-5593 | TGTAAAACGACGGCCAGTGAGTGGCGCMTGACGTACC | CAGGAAACAGCTATGACCYACGAGGAATTGYGGGCG |
| 2 | 5373-5691 | TGTAAAACGACGGCCAGTCACCGCSCAGAGCCTGAR | CAGGAAACAGCTATGACCTGRTTCATGTCGAACTCGGTG |
| 2 | 5447-5797 | TGTAAAACGACGGCCAGTGRGACACCGARGACTGCC | CAGGAAACAGCTATGACCCAGRGTGCARTAGCTGCCR |
| 2 | 5450-5800 | TGTAAAACGACGGCCAGTACACCGARGACTGCCAYGC | CAGGAAACAGCTATGACCGCGCAGRGTGCARTAGCTG |
| 2 | 5499-5830 | TGTAAAACGACGGCCAGTSGCATGGGCSAAAGAGTGG | CAGGAAACAGCTATGACCCTCGCARCCGGTCTCWGT |
| 2 | 5630-5968 | TGTAAAACGACGGCCAGTGGCAGGCYCAYTACACYACC | CAGGAAACAGCTATGACCGCCYTCRGGGAGRAAGATGA |
| 2 | 5679-5997 | TGTAAAACGACGGCCAGTCGACATGAAYCAGACCCTYGC | CAGGAAACAGCTATGACCGGGGTCCACTTGAGYGCSG |
| 2 | 573-890 | TGTAAAACGACGGCCAGTCGAGCTSATGCGCACCATY | CAGGAAACAGCTATGACCCGCARTGYTGGGCRACCT |
| 2 | 5841-6178 | TGTAAAACGACGGCCAGTCGARCCYGCCACGCTAYT | CAGGAAACAGCTATGACCGAGGGCYACCTGCTGYTCYT |
| 2 | 5925-6261 | TGTAAAACGACGGCCAGTGATTTTCCAGGGCGACGAC | CAGGAAACAGCTATGACCCGCTCYGCGCTGTARTCGTA |
| 2 | 6026-6364 | TGTAAAACGACGGCCAGTTYCCAGTGAAGCAYGTRAGCA | CAGGAAACAGCTATGACCCGCGTAGGGRGTCTGAATYTC |
| 2 | 6109-6443 | TGTAAAACGACGGCCAGTCACCARGCDATYAAGGTGCT | CAGGAAACAGCTATGACCGGTGATGACCTGGTTRGAKTARG |
| 2 | 6164-6498 | TGTAAAACGACGGCCAGTAGCAGGTRGCCCTCCTYGAC | CAGGAAACAGCTATGACCGGCTCTCCCAAATRGCARGA |
| 2 | 6342-6690 | TGTAAAACGACGGCCAGTGGARATTCAGACYCCCTACGC | CAGGAAACAGCTATGACCCGRGGCCCTCCDGAGTCR |
| 2 | 6395-6743 | TGTAAAACGACGGCCAGTCCTGCACGYRGGGCCTTT | CAGGAAACAGCTATGACCGGGCCYTGGACCAGTCYT |
| 2 | 6503-6830 | TGTAAAACGACGGCCAGTGTGCCCAAATGGCTWCYAC | CAGGAAACAGCTATGACCGGGGTTGRGGCDGYTGTG |
| 2 | 6641-6988 | TGTAAAACGACGGCCAGTACTCYAGCACCTCCGGVGAC | CAGGAAACAGCTATGACCCCARAGCCAYGAGGTCACRC |
| 2 | 6874-7199 | TGTAAAACGACGGCCAGTCTGGGGCCGCCRACYAAC | CAGGAAACAGCTATGACCGCTCRGCRTAGGTGCGCT |
| 2 | 6895-7245 | TGTAAAACGACGGCCAGTTTCCAGGCRGCCGTGGSGCGBGG | CAGGAAACAGCTATGACCATGCGCAGCARTCGGTGCC |
| 2 | 7138-7488 | TGTAAAACGACGGCCAGTAAYCAACCYGCCGGCGAC | CAGGAAACAGCTATGACCCCGTARTGCTTCCCRTGCS |
| 2 | 7159-7508 | TGTAAAACGACGGCCAGTAGGGGYATYTGGGGYAAGG | CAGGAAACAGCTATGACCGCAGYTGRTGGTGGTGGTG |
| 2 | 7302-7639 | TGTAAAACGACGGCCAGTYATCGAGACMCGCTCGGC | CAGGAAACAGCTATGACCGTGCCARTCGCTCAGGTTGT |
| 2 | 7341-7691 | TGTAAAACGACGGCCAGTCTTYGGYGCCCCTCAGGT | CAGGAAACAGCTATGACCGCTCCACRCACCAGAAGTCC |
| 2 | 737-1054 | TGTAAAACGACGGCCAGTCYACGCCCGGCTACACCC | CAGGAAACAGCTATGACCCAGCATRCGCTGRAACACGG |
| 2 | 7540-7890 | TGTAAAACGACGGCCAGTACCYTGCGCGTCGGBSAG | CAGGAAACAGCTATGACCGACAGCCCGCARATYARCC |
| 2 | 7559-7896 | TGTAAAACGACGGCCAGTACCAYCGAAACGCYAGYGAC | CAGGAAACAGCTATGACCGTRGTRGACAGCCCGCARAT |
| 2 | 7728-8053 | TGTAAAACGACGGCCAGTCACCGCRGCGAAYKCCAC | CAGGAAACAGCTATGACCSATRGGGCTCACCAGTTCYG |
| 2 | 7792-8121 | TGTAAAACGACGGCCAGTCTYAATGAYAGCTGCGGYGG | CAGGAAACAGCTATGACCTGGTCAAKRGACAGYGCGTT |
| 2 | 7901-8245 | TGTAAAACGACGGCCAGTAGTACCCRCCYACCCGMTTC | CAGGAAACAGCTATGACCGGGGKTGTACCCCTGSAGRA |
| 2 | 7951-8294 | TGTAAAACGACGGCCAGTTGGGAACTKGTCGTYCTTACGG | CAGGAAACAGCTATGACCACCCCGGYGCAGTGCAGA |
| 2 | 8144-8494 | TGTAAAACGACGGCCAGTTGGTCCCGTGGGTYYTGATA | CAGGAAACAGCTATGACCCTGCGTGCCGTTCCAGAT |
| 2 | 8255-8603 | TGTAAAACGACGGCCAGTGCGAGGAGGCTTTCACCTAC | CAGGAAACAGCTATGACCCRGTGGGGTGGTAYTGCTTG |
| 2 | 830-1171 | TGTAAAACGACGGCCAGTGCCTGTGGACACGCGATT | CAGGAAACAGCTATGACCCTTCCACCCCTCYTGCAGC |
| 2 | 8437-8787 | TGTAAAACGACGGCCAGTGGCTTGGGRGCCTGGGTR | CAGGAAACAGCTATGACCTTGCACGACRCGCCSGCYAC |
| 2 | 8476-8819 | TGTAAAACGACGGCCAGTATCTGGAACGGCACGCAG | CAGGAAACAGCTATGACCGCGTGTTGCAGAACGGRTGT |
| 2 | 8632-8972 | TGTAAAACGACGGCCAGTAGCGACGCRGCSTGCTGGGG | CAGGAAACAGCTATGACCARTGGCGYTGGCAAACCG |
| 2 | 8653-8994 | TGTAAAACGACGGCCAGTTTYCYCACCGACACCGTGAT | CAGGAAACAGCTATGACCACGAGCCGCGAACAGTCR |
| 2 | 8827-9175 | TGTAAAACGACGGCCAGTCAACTCGAGGTYCAGGTCCC | CAGGAAACAGCTATGACCCCACTCAGGCATTTCGACRG |
| 2 | 8917-9249 | TGTAAAACGACGGCCAGTGGGAGCCCRAAYTGTCAC | CAGGAAACAGCTATGACCGGGCGRACYGTCTTRAACTT |
| 2 | 9135-9483 | TGTAAAACGACGGCCAGTCGGACCGTACGGCCAYGC | CAGGAAACAGCTATGACCCTCGCGCGRTCGCTCTCR |
| 2 | 9155-9463 | TGTAAAACGACGGCCAGTCYGTCGAAATGCCTGAGTGG | CAGGAAACAGCTATGACCCCCGCARCTCACYTGGTAGG |
| 2 | 9400-9738 | TGTAAAACGACGGCCAGTCCTGGGAARTTCGTCACCG | CAGGAAACAGCTATGACCCTAGTGGGCTRRTGCGGGTT |
| 2 | 980-1328 | TGTAAAACGACGGCCAGTACCTTGTCCAYCTCGCYGAR | CAGGAAACAGCTATGACCTRATGGCGTCCCARATSGTG |

* The first number corresponds to the starting RV nucleotide of the forward primer and the second number corresponds to the end RV nucleotide for reverse primer.

^ The first 18 nucleotides of the primer sequences are the M13 tags.
